# Sprayable superhydrophobic nano-chains coating with continuous self-jumping of dew and melting frost

**DOI:** 10.1038/srep40300

**Published:** 2017-01-11

**Authors:** Shanlin Wang, Wenwen Zhang, Xinquan Yu, Caihua Liang, Youfa Zhang

**Affiliations:** 1 Jiangsu Key Laboratory of Advanced Metallic Materials, School of Materials Science and Engineering, Southeast University, Nanjing 211189, P. R. China; 2 School of Energy and Environment, Southeast University, Nanjing 210096, P. R. China

## Abstract

Spontaneous movement of condensed matter provides a new insight to efficiently improve condensation heat transfer on superhydrophobic surface. However, very few reports have shown the jumping behaviors on the sprayable superhydrophobic coatings. Here, we developed a sprayable silica nano-porous coating assembled by fluorinated nano-chains to survey the condensates’ dynamics. The dewdrops were continuously removed by self- and/or trigger-propelling motion due to abundant nano-pores from random multilayer stacking of nano-chains. In comparison, the dewdrops just could be slipped under the gravity effect on lack of nano-pores coatings stacked by silica nano-spheres and nano-aggregates. More interestingly, the spontaneous jumping effect also occurred on micro-scale frost crystals under the defrosting process on nano-chains coating surfaces. Different from self-jumping of dewdrops motion, the propelling force of frost crystals were provided by a sudden increase of the pressure under the frost crystal.

Inorganic nano-particles paint has been ubiquitously used in production and life due to simple operation and outstanding designability. In recent years, superhydrophobic coatings have been developed by one-step spray-coated inorganic nano-particles suspension, such as TiO_2_^1^ and SiO_2_^2^. Currently, nano-particles superhydrophobic coatings were mainly prepared by followed two methods: 1) fluorinated nano-particles were assembled into superhydrophobic coating by spray, spin or dip methods^1^,^2^,^3^; 2) as same as the traditional fabrication method of superhydrophobic films, the as-achieved nano-particles coating was modified by polymer with low surface free energy^4^,^5^. These superhydrophobic nano-particles coatings promise a great application potential in future because it could be extensively expanded and functionalized on various substrates. However, it is still challenging to fabricate sprayable superhydrophobic coatings with micro-scale dew and melting frost continuous self-propelled jumping effect.

In real-world, formation and accumulation of dew and frost cannot be avoided on cold surfaces. As a consequence, the heat transfer efficiency was significantly impeded in energy conversion fields^6^,^7^. In order to effectively restrain dewing and frosting on the solid-gas interface, water repellent films were largely designed because of high potential barrier for condensate nucleation^8^,^9^,^10^,^11^. In recent years, **condensed dewdrops self-ejecting (CDSE)** effect was mainly developed on superhydrophobic surfaces to significantly enhance the heat transfer and dewing/frosting resistance^12^,^13^,^14^,^15^,^16^. Evidences from the literatures^17^,^18^ suggest that rough nanostructure of the surfaces with CDSE effect were first constructed by two-tier micro-nano composite structures. Subsequently, the CDSE phenomena were also observed on treated metal surfaces, such as schistose porous Al(OH)_3_ films^19^,^20^, oxidized Cu surface^21^,^22^,^23^,^24^ and electrodepositing nano-particles^25^,^26^,^27^,^28^,^29^. Then, nano-arrays, assembled by carbon nano-tubes^30^, ZnO nano-needles^31^,^32^, polymer nano-cone^33^ or Al nanorods^34^,^35^, were also reported to obtain the CDSE surfaces. In order to make superhydrophobic surfaces more widely used on various substrates, a commercial coating agent was poured on the smooth silicon wafer to characterize the CDSE effect^36^. Therefore, it is necessary to develop a simple yet versatile approach for assembly of CDSE surfaces^37^,^38^ for application in energy saving.

Based on the principle of CDSE effect, if two or more micro-drops coalescence, the gain in surface energy was partially transferred into kinetic energy. CDSE behaviors were triggered when the adhesion froce is low enough between the droplet and the surface. Herein, we propose that CDSE surfaces may be assembled by catenulate silica nano-particles, which is inspired by carbon nano-tubes^30^ and cerium oxide porous nanoparticle^27^. To confirm this opinion, we fabricated the superhydrophobic coating by one-step spray methods from nano-chains, nano-spheres and nano-aggregates paints respectively for comparison of condensation performances. For another, self-propelling motion of isolated droplets or particles also could be triggered by unbalanced pressure^39^,^40^,^41^. Thus, we designed a frosting-defrosting process to achieve local high-pressure under the frost crystal on the nano-chains coating for observation of the **melting frost self-ejecting (MFSE)** motion.

## Results and Discussions

### Coatings preparation

**Figure 1:**
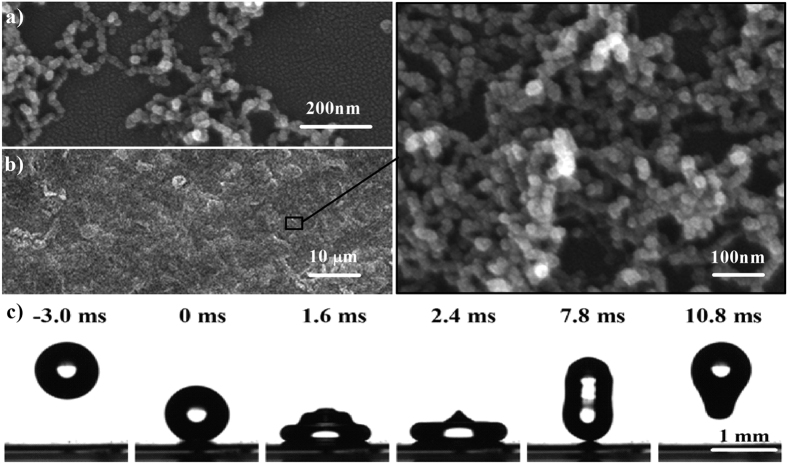
Microscopy morphology of F-chains coating was characterized by Field-emission scanning electron microscopy (FESEM) in the (**a**) initial and (**b**) final stage. (**c**) Water droplet bouncing test. 5 μL water droplet with diameter of 1.1 mm perpendicularly impacts by free fall from 10 cm high, the impact velocity is about 1.4 m/s. The contacting time between the droplet and the surface from encounter to separation is about 7.8 ms and the droplet completely leaved the surface without wetted, contaminated, penetrated or damaged.

Sprayable paint of **fluorinated silica nano-chains (F-chains)** were fabricated according to literature^2^. A spray painting apparatus equipped with S-130 airbrush (U-STAR, nozzle diameter of 0.3 mm) and U-601G gas compressor (U-STAR, 20 psi) was used to spray the paint on cleanly substrate for acquisition of superhydrophobic surfaces. The airbrush was moved (about 0.1 m·s^−1^) maintaining about 10 cm distance and according to an S-style spray route from top to bottom. This process could control the thickness (5–10 μm) of the coating by spray-steps. Figure 1a displays that the F-chains coating was assembled by single-layer reticulated-like structure in the initial stage, which is interlocking by a nature of soft string-of-pearls structure with diameter of ~15 nm and length of ~80 nm (More details see Supporting Information, Figure S1). After spray painting, nano-porous foam-like coating was obtained by random multilayer stacking of nano-chains (Fig. 1b)). In contrast, we also fabricated the superhydrophobic paints using mono-dispersed silica nano-spheres (F-spheres) or fumed silica nano-aggregates (F-aggregates) to replace nano-chains. Figure S2a–c shows that the F-spheres with diameter of ~100 nm (More details see Supporting Information, Figure S1) only developed a close-packed stacking structure. Similarly, the F-aggregates coating with small amounts of nano-pores was stacked by the irregular cluster with size of ~100 × 200 nm (More details see Supporting Information, Figures S1 and S2e–f)). All of above paints were sprayed on various hard and soft substrates to fabricate superhydrophobic surfaces and their superhydrophobicity has no significant difference if measured by macroscopic water droplet^2^,^3^,^5^. For F-chains coating, **static water contact angles (SCAs)** of over 160° and **roll-off angles (RAs)** of under 4° were demonstrated (More details see Supporting Information, Figure S3). Moreover, the substrates could repel the impacting water droplet by bouncing off due to the Cassie-Baxter state of superhydrophobicity^42^,^43^ (Fig. 1c, More details see Supporting Information, Video S1). However, it still needs further investigation whether the micro-drops can maintain the Cassie-Baxter state on the surfaces with above different coatings.

### Condensation performance of dewdrops

**Figure 2:**
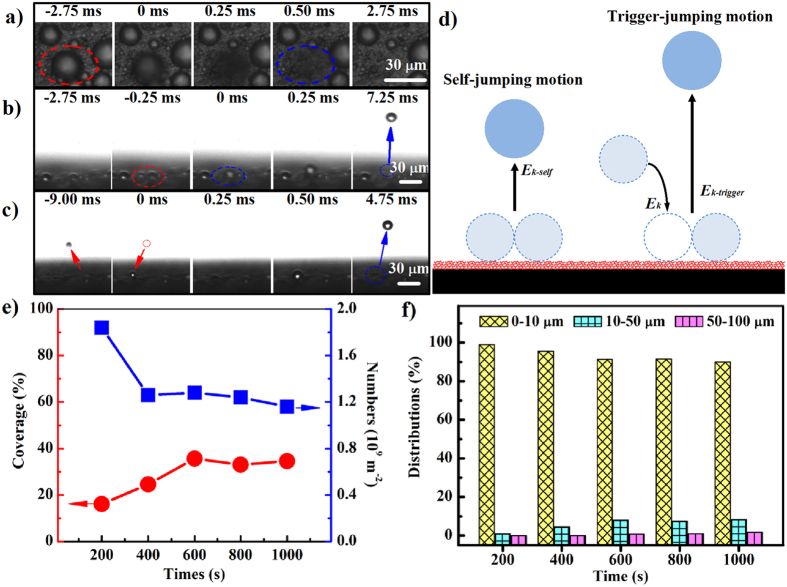
Typical CDSE effect on the F-chains superhydrophobic surface placed horizontally were examined by (**a**) top-view and (**b,c)** side-view. (**a**) The coalescence and departure behavior of several droplets with different sizes of 21.2 μm, 12.4 μm and ~5.3 μm. (**b**) The droplet on the right growing to 11.5 μm and then merging with the left droplet with a diameter of 12.5 μm, the merged drop with 15.9 μm bouncing off the surface above 90 μm by a initial velocity of ~0.1 m/s. (**c**) A jumped droplet with 8.4 μm falling back to the surface from 48.8 μm to impact and merge the other droplet, the merged again droplet with 14.7 μm continuous bouncing away above 90 μm by a initial velocity of ~0.1 m/s. (**d**) Schematic illustrations of self- and trigger-jumping motion on F-chains surface. As shown previously^23^,^31^,^36^, E_*k*_ = E _*s*_− E_*v*_ − E_*i*_ + ∆E_*k*_, where E_*k*_ is the kinetic energy of the jumping micro-drop, E_*s*_ is the released surface energy, E_*v*_ is the viscous flow-induced energy dissipation, E_*i*_ is the interfacial adhesion-induced energy dissipation and ∆E_*k*_ is the kinetic energy of the moving droplet. When ∆E_*k*_ = 0, E_*k*_ = E_*k-self*_. ∆E_*k*_ > 0, E_*k*_ = E_*k-trigger*_. (**e**) The condensed micro-drops coverage and number of unit changed with time on F-chains coating. After condensation 600 s, both the coverage and numbers were also came into a dynamic balancing. (**f**) Micro-drop number distributions of dewdrops against time and multi-repetition condensation on F-chains surfaces. The size and distribution tend to achieve dynamic balancing after condensation 600 s. (Cooling stage temperature *T*_*c*_ = 5 °C, ambient temperature *T*_*a*_ = 25 °C and relative humidity *HR* = 50%, dew-point temperature *T*_*d*_ = 13.86 °C. All statistics were carried out under a low-magnification images with 2 × 2 mm. Here, we partitions the images as 100 regions with 200 × 200 μm and then random selection 10 ones to measure the average value of size and number.)

To investigate the condensation performance on the F-chains surface, we used conventional condensation test on a cooling stage at the controlled environment. Here, condensation tests were carried out on polished copper sheet with size of 25 × 25 mm after spray-coated by nano-particle coating. Figure 2a shows the coalescence and self-expulsion processes of several adjacent condensed micro-drops without any external force (More details see Supporting Information, Video S2). Two types of CDSE phenomena were observed: 1) the dewdrops were firstly nucleated on the tops of F-chains coating and then growing by the Cassie-Baxter mode; the two or more droplets finally jumped out their original position after contacting and merging (Fig. 2b, More details see Supporting Information, Video S3) due to the vapor emitted by neighboring droplets^41^; 2) a collision could be triggered between falling droplet after ejected and static dewdrop (Fig. 2c), More details see Supporting Information, Video S4). In the past, we aways focus on the self-jumping motion on the superhydropobic surface, but pay no attention to the continuous trigger-jumping motion as a important role for rapidly propellion of dewdrops, which is significant to more efficaciously maintain the surface as dry as possible at the condition of dewing. Intelligibly, apart front excess surface energy, the trigger-jumping effect will get more energy from surplus kinetic energy after collisions (Fig. 2d)^23^. Thus, the trigger-jumping behavior not noly formed higher droplet velocity for accelerated removal of dewdrops, but also expedited increase of the size of droplet to easily depart from the surfaces.

Based on the continuous and periodical CDSE function on F-chains coating (More details see Supporting Information, Video S5), we also examines the statistical parameters of condensed micro-drops on a horizontal cooling stage. The coverage, numbers of unit and distribution of dewdrops on F-chains surfaces charged with times were shown in Fig. 2(e) and (f). The nucleation and growth of the condensed droplets were mainly developed at 0–200 s. At 200 s, the dimension of droplets is below 5 μm with low coverage rate (lower than 20%) and the CDSE behaviors were only detected in tiny region. When the size is growing to 10–50 μm after 400 s, the CDSE phenomena have significantly increased. After 600 s, the CDSE effects reached the prosperous stage and various statistics of dewdrops keeping in a dynamic equilibrium. However, condensation test shows that the CDSE phenomenon was not found on the F-spheres and F-aggregates coatings although they have excellent superhydrophobicity. To further demonstrate the CDSE characteristic, we examined the morphological changes of dewdrops by environmental scanning electron micrograph (ESEM). Figure S4(a) suggests that the adjacent droplets with diameters of ~10–50 μm on the F-chains coating deviated from their original position to out of sight and presented a dry areas. Subsequently, new condensed micro-drops will nucleate again on the same location after previous droplets vanish, then the merged and jumped behaviors were surveyed again when new droplets grew to ~27 μm. Such periodical process of nucleating, growing, merging and disappearing on the same place will sequentially guarantee the statistical parameters of condensed micro-drops in dynamic equilibrium. However, we only caught the continuous consolidation process with non-jumping effect on the F-spheres and F-aggregates coatings. Figure S4(b) performed that the diameters of a dewdrop constantly increase from 18 μm to 120 μm on the F-aggregates coating. The results suggest that nano-porous structure formed by interlaced nano-chains will conduced to enhance CDSE effect on superhydrophobic surface.

### MFSE motion of frost crystal

Evaporation could provide the propelling force for spontaneous movement of droplets or particles under a condition of unbalanced air pressure. In the past, the environment with vacuum^39^ and/or high-temperature^40^ was used to build the condition of low-vacuum and/or low-humidity. Inspired by this, we attempted to fabricate local environment with low-vacuum and low-humidity by a frosting process. It is well known that the frosting could not be completely restrained under super-cooling conditions^44^ although the CDSE behaviors provided a highly effective method to suppress it^16^. The environment with low-vacuum and/or low-humidity would be obtained under the frost crystal once it was formed on the nano-porous films. Now, partial vaporization was released from the frost crystal to keep the pressure balance. Then, if we rapidly elevated the temperature of substrate, the accumulated low-temperature vapor was quickly expanded to form a high-pressure for provision of propelling force.

**Figure 3:**
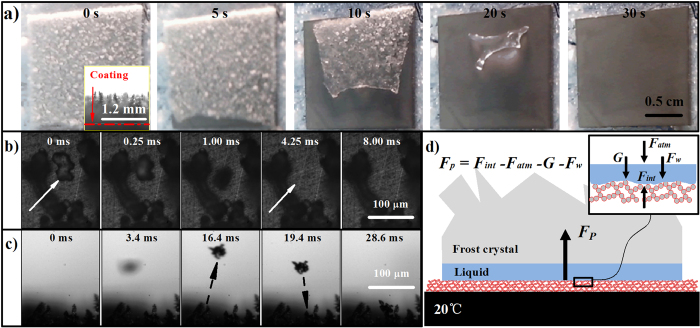
(**a**) The frost morphology evolution during the defrost process on the F-chains surface placed horizontally. Inset: The profile of frost on the F-chains surface under −20 °C after 20 min, the high of the frost is about 1.2 mm. Typical MFSE effects on the F-chains coating were examined by (**b**) top-view and (**c**) side-view. (**b**) The white solid arrow shows an isolated frost crystal rapidly deviating from their original location and then disappeared in sight. (**c**) The black dotted arrow displays the movement track of the melting frost crystal under defrosting process. (More details see Supporting Information, Video S6 and S7). (**d**) Schematic illustrations of the self-propelling mechanism of the frost crystal on the F-chains surface placed horizontally under defrosting process.

Here, we performed the frosting-defrosting test on a cooling stage. We firstly reduced the temperature of the cooling stage to −20 °C for 20 min to insure the frost covered all the cooled surfaces. And then the temperature was rapidly increased (5 °C/s) to 20 °C for adequate defrosting. Figure 3(a) catches the defrosting process on the F-chains coating. The frost crystals were fast removed less than 30 seconds and leaving a dry surface without any evidence of damage. As shown previously^38^, a lubricating layer could be formed between the frost and the superhydrophobic surface before the shrinkage deformation of the frost crystal. To further demonstrate the existence of lubricating layer, we examined the dynamics of defrosting on the micro-scale. As expected, the MFSE behavior was captured on the F-chains film during the defrosting stage. Figure 3(b) displays that an irregular frost crystal with sectional dimension is ~50 × 60 μm completely departed from the substrate with self-rotation and disappeared in perspective after 8 ms. The other one with diameter of ~31.8 μm jumping up from the surface by an initial velocity is 0.1 m/s without any external force (Fig. 3(c)). The frost crystal finally landed in another position from 150 μm high after 28.6 ms. Thus, we believe that the lubricating layer was constituted by transient state intermediate liquid and they released vapors due to supersaturated vapor pressure in stuffy solid interface between frost layer and nano-porous coating.

To estimate the self-propelling force (*F*_*P*_) of the frost crystal, we developed the following formula inspired by references^39^,^45^ on one-dimensional model (Fig. 3(d)).


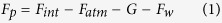


where, *F*_*int*_ and *F*_*atm*_ are the force respectively from internal and atmospheric pressure (*P*_*int*_ and *P*_*atm*_) at the bottom and top of the frost crystal, *F*_*int*_ = *P*_*int*_*A, F*_*atm*_ = *P*_*atm*_*A, A* is the solid-liquid contact area, G is the gravity and *F*_*w*_ is wetting force from viscosity. Under the standard condition, we obtained the internal pressure *P*_*int*_ = *T*_*2*_*P*_*0*_/*T*_*1*_, *T*_*1*_ and *T*_*2*_ is the absolute temperature of the cooling stage at frosting and defrosting process, *P*_*0*_ is the atmosphere pressure (101.3 kPa). Thus, we gained that the internal pressure is about 117.3 kPa. Here, G could be neglected in this calculation due to *G* ≪ *F*_*w*_^45^. *F*_*w*_ could be estimated as *F*_*w*_ = 4*Aγ*_*LV*_/*r*_f_^1/2^*D*_*0*_, *D*_*0*_ is the average diameter of the projected area between the frost crystal and coating, *r*_f_ is the roughness of the F-chains coating characterized as the ratio between the solid-liquid contact area (*A*) and the projected area (*A*_*0*_). *γ*_*LV*_ is the surface tension of water at 20 °C. Letting *F*_*p*_ = 0, we can give an estimation of the minimal value of the internal pressure (*P*_*int-min*_) under the frost crystal before jumping.


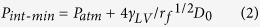


On the F-chains coating, by substituting appropriate values that *P*_*atm*_ = 101.3 kPa, *D*_*0*_ = 20 μm, *r*_f_ = 2.7, *γ*_*LV*_ = 0.073 N/m, we can obtain *P*_*int-min*_ = 110.2 kPa.

**Figure 4:**
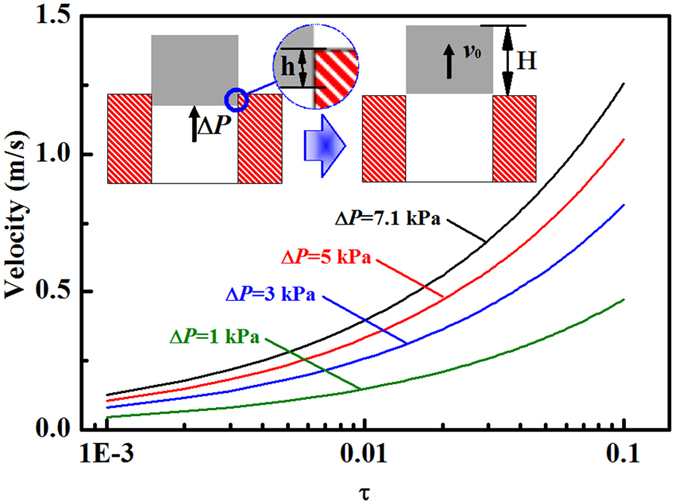
Relationship between the initial velocity and the value of τ. Inset: Schematic illustrations of the like-champagne cork motion model. Firstly, a sufficient pressure were obtained at the bottom surface of the frost crystal. Then, the pressure provides a propelling force for movement of the solid until it depart from the superhydrophobic surface. Finally, the escaped frost crystal gains the initial kinetic energy from the locomotion process before the vapor could diffuse from some aperture. Here, we assume the self-propelling pressure keeps invariant and the internal vapor will be diffused immediately before and after the frost crystal leave the coating surface, respectively.

To further discuss the relationship between initial velocity *v*_*0*_ and the self-propelling pressure (∆*P* = *P*_*int*_ − *P*_*int-min*_), we propose the like-champagne cork motion model (Fig. 4). The vapor were accumulated at the bottom surface of the frost crystal, a sudden increase of pressure provides the initial kinetic energy (*E*_0_ = *ρAHv*_0_^2^/2) of the moving solid, *ρ* and *H* is the mass density and height of the frost crystal. We propose that the frost crystal will lost the propelling pressure when it leave the nano-porous coating after motion *h* distances (∆*E*_*k*_ = ∆*PAh*). On the basis of the law of conservation of kinetic energy (*E*_0_ = ∆*E*_*k*_), we could write the dynamic equation as


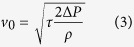


where, τ is defined as the ratio of *h* and *H, ρ* = 0.9 g/cm^3^ is the density of the frost crystal. Figure 4 plots the changing tendency of *v*_*0*_ with the increase of the τ from 0.001 to 0.1 under the self-propelling pressure of 1, 3, 5, and 7.1 kPa.

## Conclusions

In this paper, we prepared superhydrophobic coatings by spray-coating silica paint to investigate micro-scale dew and melting frost continuous spontaneous jumping behaviors. F-chains coating with nano-porous structure shows continuous micro-drops motion behaviors. Similar to previous reports, the CDSE effects were mainly observed when the size of dewdrops is growing to 10–50 μm. Over 80% of micro-drops maintaining below 10 μm on the F-chains coating even after condensation for 1000 s. However, the CDSE effects were not found on the F-spheres and F-aggregates silica coating due to lack of nano-pores. This striking property shows that construction of nano-porous is an effective mean to reduce the solid-liquid adhesion to guarantee the CDSE motion after merging. For another, the spontaneous jumping behaviors were not noly triggered by the release of excess surface energy, but also promoted by unbalanced air pressure. We successfully designed a frosting-defrosting test to control the internal pressure under the frost crystal for expression of MFSE motion. The micro-scale frost crystal with size of ~50 μm deviated from their original position without any external force. These findings are significant to develop novel feasible superhydrophobic surfaces for effective elimination of micro-scale dew and melting frost, which could be extensively applied in the heat transfer equipment and self-cleaning surface.

## Methods

### Materials

SiO_2_ sol containing with 15 wt.% SiO_2_ nano-chains or 30 wt.% mono-disperse SiO_2_ nano-spheres were referred by Nissan Chemical Industries, Ltd. Fumed SiO_2_ nano-aggregates were provided by Wenhuachem co., Ltd. 1 H, 1 H, 2 H, 2H-perfluorodecyltriethoxysilane (PFDTES) were purchased from Sikang New Material Co., Ltd. Absolute ethyl alcohol (EtOH), deionized water, ammonium hydroxide (28%), tetraethyl orthosilicate (TEOS), acetone, n-butyl acetate, and other chemical reagents were purchased from Sinopharm Chemical Reagent Co., Ltd. To remove oil contamination and oxidation film on the surface of substrate, copper sheets were orderly immersed with ultrasonic for 10 min under acetone, hydrochloric acid solution (2 mol·L^−1^), deionized water, and EtOH. Glasses, ceramic, and polyvinyl chloride (PVC) substrates were washed by detergent, deionized water and EtOH, successively. Finally, all the above substrates were dried by blower with cold air. Other substrates were used fresh samples directly, such as wood, polyethylene terephthalate (PET), paper, and cloth.

### Characterizations

The microstructure of sample surfaces were observed by field-emission scanning electron microscopy (FESEM) and atomic force microscope (AFM), which was conducted with Sirion and Dimension ICON instrument, respectively. Static water contact angels (SCAs) and roll-off angels (RAs) were observed at room temperature on an OCA 15Pro contact angle meter to characterize the hydrophobicity of films. Condensation and frosting states of the horizontal surface in different times were vertically downward collected by a JSZ6S type stereo microscope. Optical photograph of condensed micro-drops self-ejecting (CDSE), melting frost self- ejecting (MFSE) phenomena and water droplet bouncing test were captured by a Photron FASTCAM Mini UX100 type high speed camera equipped with Navitar 6000 zoom lens.

## Additional Information

**How to cite this article**: Wang, S. *et al*. Sprayable superhydrophobic nano-chains coating with continuous self-jumping of dew and melting frost. *Sci. Rep.* **7**, 40300; doi: 10.1038/srep40300 (2017).

**Publisher's note:** Springer Nature remains neutral with regard to jurisdictional claims in published maps and institutional affiliations.

## Supplementary Material

Supporting Information

Supplementary Video S1

Supplementary Video S2

Supplementary Video S3

Supplementary Video S4

Supplementary Video S5

Supplementary Video S6

Supplementary Video S7
