# Supplementary material for: Sprayable superhydrophobic nano-chains coating with continuous self-jumping of dew and melting frost
Source: Sci Rep. 2017 Jan 11;7:40300. doi: 10.1038/srep40300 (PMC5225496; doi:10.1038/srep40300)
Supplement: Supporting Information [file srep40300-s1.doc]

**Supporting Information**

**Sprayable superhydrophobic nano-chains coating with continuous self-jumping of dew and melting frost**

*Shanlin Wang^a^, Wenwen Zhang^a^, Xinquan Yu^a^, Caihua Liang^b^, Youfa Zhang^a,*^*

^a^ Jiangsu Key Laboratory of Advanced Metallic Materials, School of Materials Science and Engineering, Southeast University, Nanjing 211189, P. R. China

^b^ School of Energy and Environment, Southeast University, Nanjing 210096, P. R. China

*Corresponding author. E-mail: yfzhang@seu.edu.cn;

**KEYWORDS：condensation; self-ejection; spray coating; silica nano-chains; superhydrophobicity**


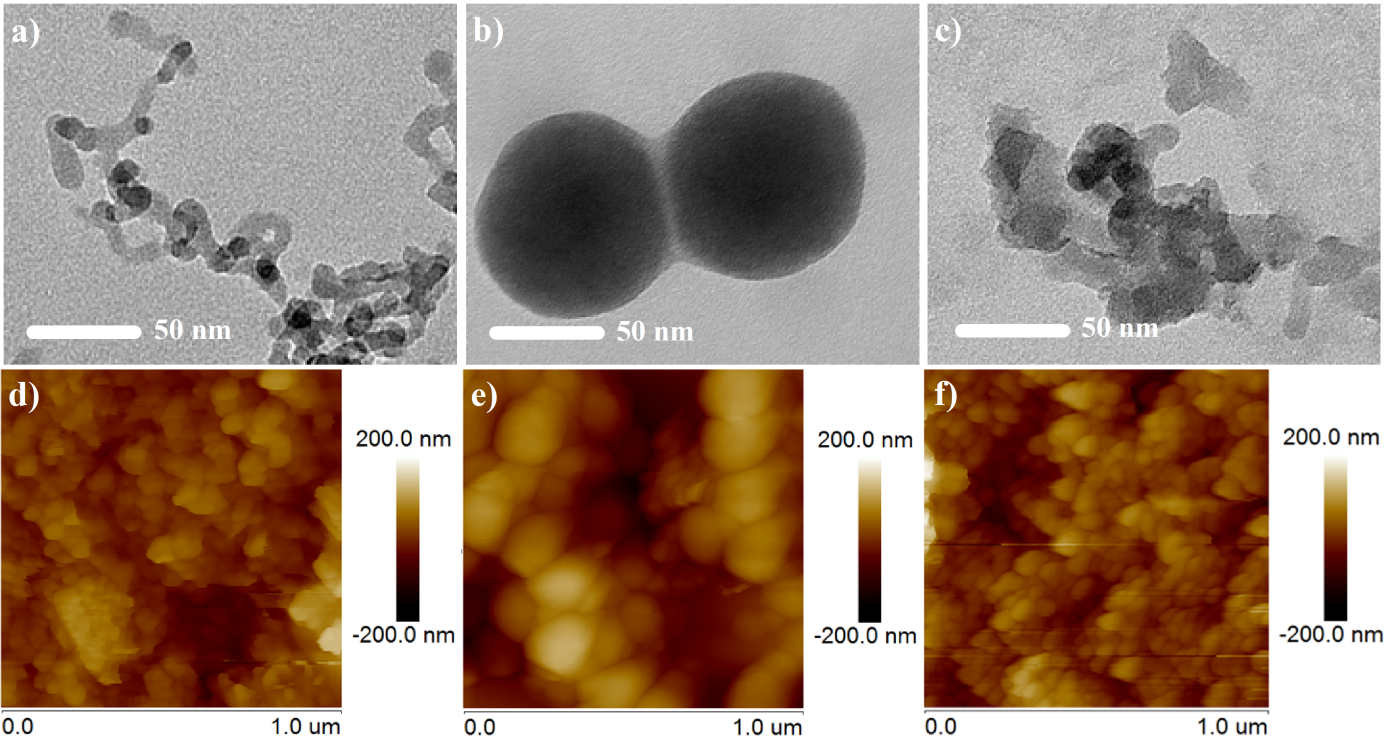


**Figures S1** Microscopy morphology of **a)** F-chains, **b)** F-spheres and **c)** F-aggregates paints were characterized by transmission electron microscope (TEM). And the coating from **d)** F-chains, **e)** F-spheres and **f)** F-aggregates were surveyed by **atomic force microscope (AFM)**. **a, d)** Ubiquitous interlocking was easily caught between in nano-chains with diameter ~10-20 nm in the F-chains paint. **b, e)** The size of the nano-spheres are maintaining in ~100 nm. Sometimes, large-size particle cluster were also combined by two or more nnao-spheres. **c, f)** The aggregates with size ~100 × 200 nm were assembled by close-packed nano-particle with diameter ~20-30 nm.


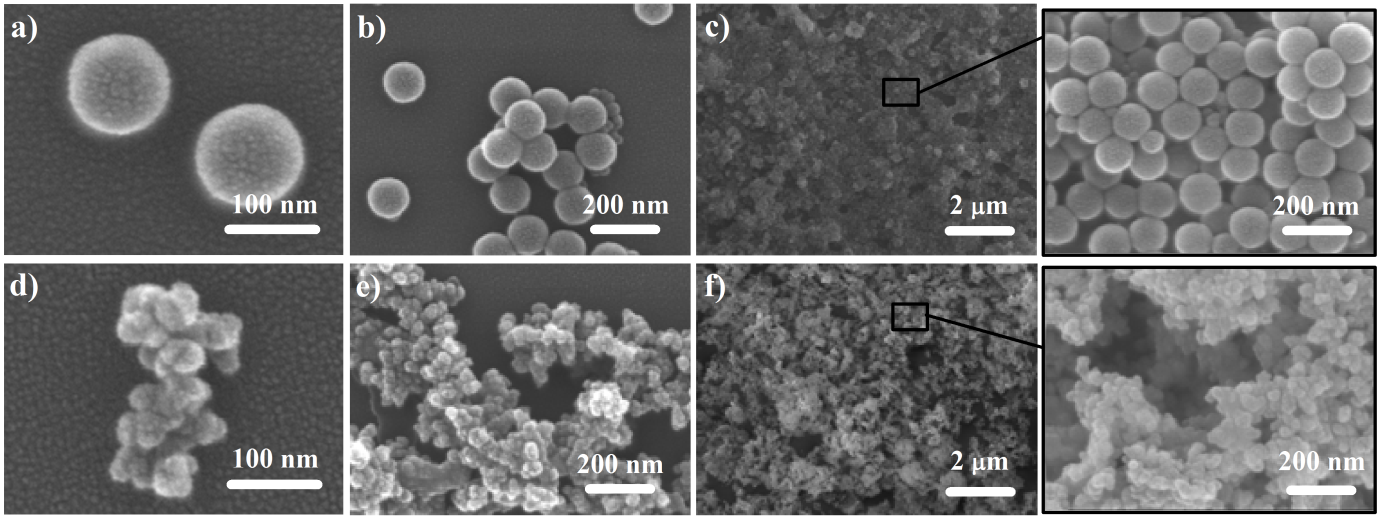


**Figures S2** Field-emission scanning electron microscopy (FESEM) images characterizing the microscopy morphology of the **a-c)** F-spheres and **e-f)** F-aggregates coatings under different assembly process at **a, d)** initial, **b, e)** middle and **c, f)** final stage.


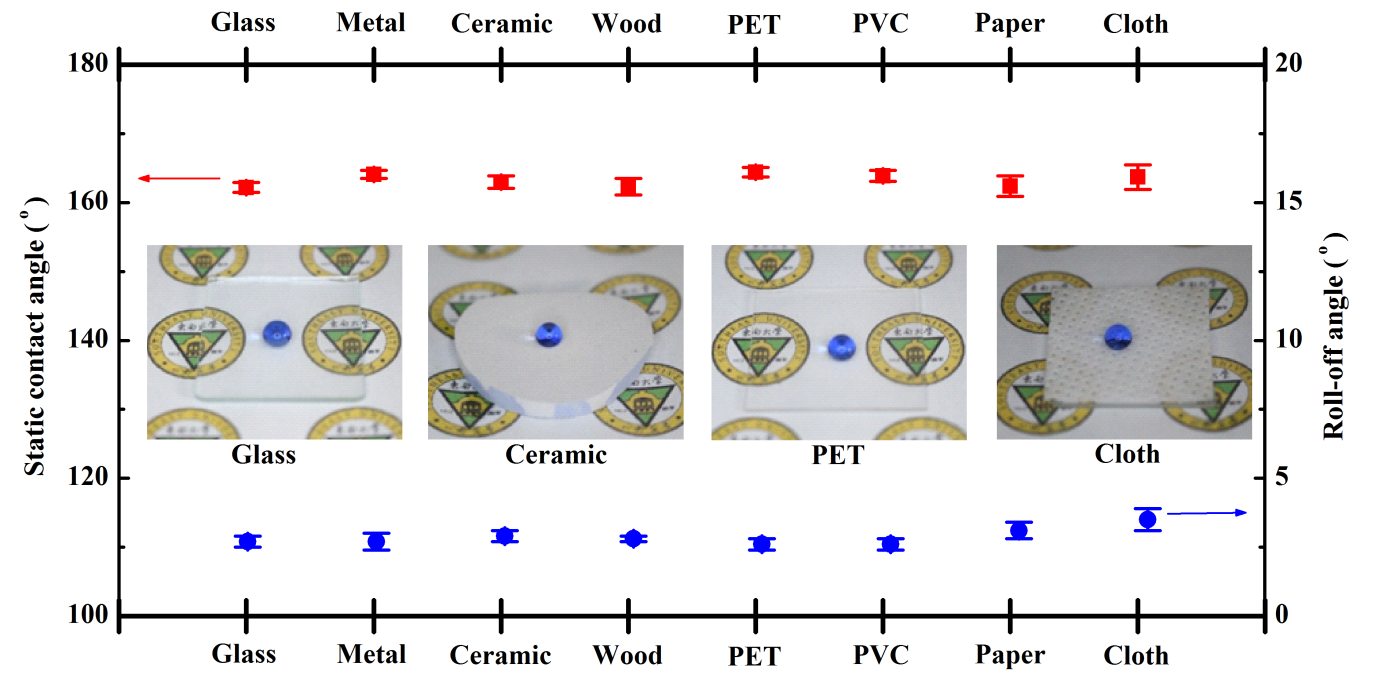


**Figures S3** Static contact angles (SCAs) and roll-off angles (RAs) of 5 µL water droplets on the F-chains surfaces with various hard and soft substrates. Inset: Optical images of water droplet dyed by methyl blue on the F-chains surfaces with different substrates. The inherent color of substrates has not yet been covered by the superhydrophobic surface.


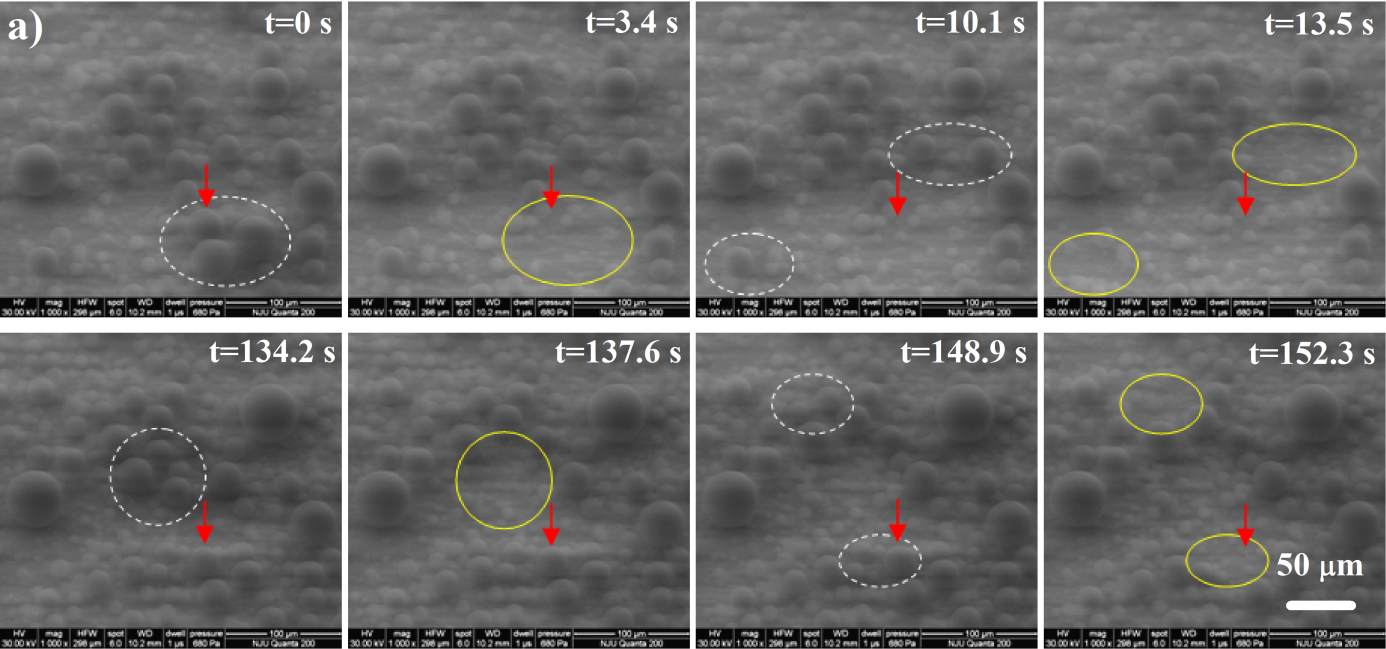


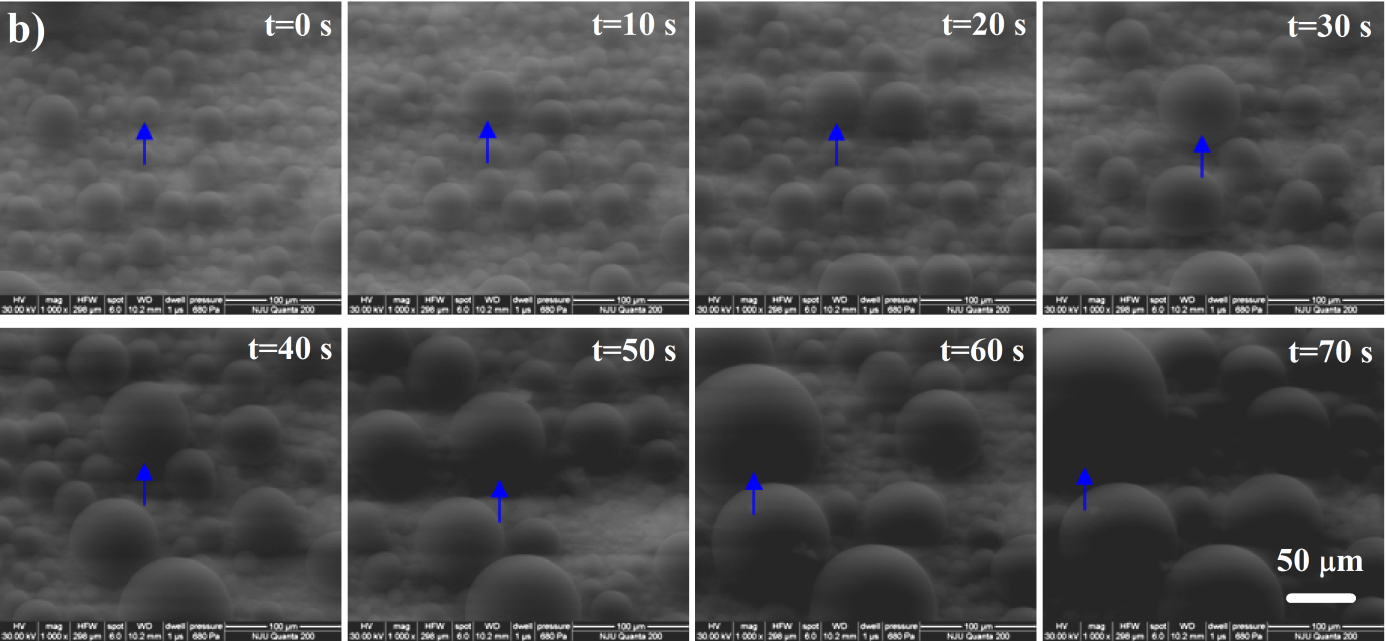


**Figure S4** Environmental scanning electron micrograph (ESEM) images of condensed droplets dynamic on the **a)** F-chains and **b)** F-aggregates superhydrophobic surface. **a)** Spherical condensed micro-drops continuously bounced off the initial position with adjacent one, two or three droplets. White dotted circles highlight areas of the surface with droplets just before merging and jumping, while yellow solid circles highlight the surface after droplet jumping. Red arrow tagged a process of droplet repeatedly bounced from same location. The droplet with diameter of 35 μm fleeing the horizon by coalesced with the others two drops with diameters of 39 μm from 0 s to 3.4 s. The new nucleation droplet grew to 27 μm after 148.9 s on the same place and then walking away again. **b)** Blue arrow displayed the spherical condensed micro-drops rapidly growing and mergening in the original location. After 70s, the size of the dewdorps is growing from 18 µm to 120 µm. (Vapor pressure *P_v_* = 680 Pa, cooling stage temperature *T_c_* = 5 ℃, saturation *S* = 1.05).

**Videos list**

**Video S1** Water droplet bouncing test. 5 µL water droplet with diameter is about 1.1 mm perpendicularly impacts by free fall from 10 cm high, the impact velocity is about 1.4 m/s. The contacting time between the droplet and the surface from encounter to separation is about 7.8 ms and the droplet completely leaved the surface without wetted, contaminated, penetrated or damaged.

**Video S2-S5** The CDSE effects on F-chains surfaces were captured by high speed camera at top- and side-view during the condensing process.

**Video S6-S7** The MFSE behaviors on F-chains surfaces were shot by high speed camera at top- and side-view during the defrosting process.
